# CUMULATIVE DIS/ADVANTAGE IN HEALTH AS AN INTERGENERATIONAL PROCESS

**DOI:** 10.1093/geroni/igz038.1563

**Published:** 2019-11-08

**Authors:** Kim Shuey

**Affiliations:** Department of Sociology, :University of Western Ontario, London, Ontario, Canada

## Abstract

Empirical applications of cumulative dis/advantage primarily focus on accumulative processes within an individual life course as a mechanism generating growing intracohort inequality with age. However, adult health reflects the accumulation of exposures to advantages and disadvantages across the individual life course as well as the transmission of resources and practices across generations within one’s family of origin, which forms the foundation from which children are launched and inequality is reproduced. In analyses using two panel studies (PSID and Add Health), we integrate literature on the early-life origins of health with the concept of linked lives and the intergenerational transmission of resources to examine the relationship between parents’ health and the health of their children in adulthood. Results indicate an intergenerational persistence in health and demonstrate the importance of looking beyond the individual life course to better understand cumulative dis/advantage in health as a process operating across generations within families.

